# Extrusion 3D printing of a multiphase collagen‐based material: An optimized strategy to obtain biomimetic scaffolds with high shape fidelity

**DOI:** 10.1002/app.53593

**Published:** 2023-01-09

**Authors:** Giorgia Montalbano, Andrea Roberto Calore, Chiara Vitale‐Brovarone

**Affiliations:** ^1^ Department of Applied Science and Technology Politecnico di Torino Torino Italy; ^2^ Fondazione Istituto di Ricerca Pediatrica Città della Speranza Padova Italy

**Keywords:** 3D printing, additive manufacturing, biopolymers, composites, multiphase systems, type I collagen

## Abstract

Extrusion printing represents one of the leading additive manufacturing techniques for tissue engineering purposes due to the possibility of achieving accurate control of the final shape and porosity of the scaffold. Despite many polymeric materials having already been optimized for this application, the processing of biopolymer‐based systems still presents several limitations mainly ascribed to their poor rheological properties. Moreover, the introduction of inorganic components into the biomaterial formulation may introduce further difficulties related to system homogeneity, finally compromising its extrudability. In this context, the present study aimed at developing a new multi‐phase biomaterial ink able to mimic the native composition of bone extracellular matrix, combining type‐I‐collagen with nano‐hydroxyapatite and mesoporous bioactive glass nanoparticles. Starting from a comprehensive rheological assessment, computational‐fluid‐dynamics‐based models were exploited to describe the material flow regime and define the optimal printing process planning. During printing, a gelatin‐based bath was exploited to support the deposition of the material, while the gelation of collagen and its further chemical crosslinking with genipin enabled the stabilization of the printed structure, characterized by high shape fidelity. The developed strategy enables the extrusion printing of complex multi‐phase systems and the design of high‐precision biomimetic scaffolds with great potential for bone tissue engineering.

## Graphical abstract

A multiphase biomaterial ink combining type‐I‐collagen with nano‐sized mesoporous bioactive glasses and nanohydroxyapatite was successfully designed to mimic the natural composition of bone. Based on rheological data and computational‐fluid‐dynamic models, the printing set‐up was optimized to obtain scaffolds characterized by high printing fidelity and complex geometries, where the final mechanical stability was further enhanced by a chemical genipin crosslinking method.

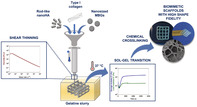

## 1 INTRODUCTION

In recent decades, tissue engineering is emerging as a strategic alternative to meet the growing demand for rapid and effective tissue regeneration following injuries and pathological conditions. The correct choice of biologically inspired biomaterials and fabrication processes is therefore considered a key factor in designing engineered constructs that mimic the complex nano‐, micro‐ and macro‐structures, as well as the natural mechanical and biological properties of native tissues.^1^, ^2^

At present, additive manufacturing is the only approach that enables the fabrication of 3D scaffolds with a highly controlled internal structure and pore morphology, while ensuring improved reproducibility, time savings and cytocompatibility.^3^, ^4^

With the aim of expanding the range of processable biomaterials and enabling the production of larger scaffolds, extrusion‐based technologies, also known as robotic dispensing, are the most widely used, promising, and versatile 3D printing techniques. However, compared to laser‐induced and inkjet printing, the final resolution of scaffolds is normally significantly lower, and strongly depends on the specific rheological properties of the selected materials.^5^ Despite the use of these technologies dating back to more than two decades, the level of detail and precision required to design complex constructs has only been recently addressed, attracting considerable efforts to design biomimetic scaffolds capable of reproducing the natural characteristics of different tissues in various respects.^6^

Regardless of the printing approach considered, the final shape fidelity and print resolution are strongly influenced by the visco‐elastic properties of the inks developed. Accordingly, shear‐thinning and fast shear recovery are specific rheological properties required to enable the correct extrusion and stabilization of post‐printed structures in the case of extrusion‐based technologies.^7^, ^8^

Moreover, the individual fabrication platform and the overall printing set‐up strongly influence the characteristics of the resulting constructs. Consequently, a detailed assessment of the printability and processability of the material is crucial to define the best conditions and parameters, ensuring reproducible and reliable results.

In this context, computational fluid dynamics (CFD) models can support the identification of the most promising printing set‐up by combining the rheological properties of the developed biomaterial with the features of the selected printing systems. Considering the current trial‐and‐error approach in 3D printing,^9^ CFD simulations allow to screen feasible printing conditions and to estimate the printing parameters in advance, therefore enabling the reduction of potential material waste and the optimization of the overall workflow.^10^

After assessing the actual processability of the material with the selected technique, the final print set‐up requires further optimization according to its specific physico‐chemical properties. Despite extrusion printing techniques proving their high potential in the realization of more complex structures compared to traditional manufacturing, the choice of cytocompatible inks is still limited, particularly in the case of naturally derived materials and biopolymers such as type I collagen. These biomaterials require processing under a restricted range of thermal and chemical conditions to preserve their native physico‐chemical properties, therefore narrowing the spectrum of suitable manufacturing approaches and sometimes requiring appropriate process modifications to ensure final processability.^11^, ^12^

The choice of materials is normally based on the manufacturing technique used and the nature of the tissue to be regenerated, with the possibility of modulating the material's properties through the combination with organic and inorganic phases or the addition of dispersing agents to improve the final processability.^13^

On the contrary, in the case of biomimetic constructs intended to reproduce the specific composition of the tissue, there is a strong limitation on the final material formulation, and it may therefore be necessary to adapt the manufacturing process to the material's specific characteristics.^6^, ^14^

In the field of bone‐tissue engineering (BTE), nanomaterials and 3D printing technologies can be exploited to design biomimetic constructs, considering that bone itself is characterized by a complex hierarchical architecture and a perfectly arranged nanocomposite nature.^15^ Currently, most 3D printed scaffolds for BTE are based on the use of thermoplastic polymers such as polycaprolactone (PCL) or poly‐l‐lactic acid (PLLA) possibly grafted or blended with biopolymers and bioactive molecules to increase the final biocompatibility of the system.^4^, ^16^, ^17^ Nevertheless, type I collagen, as the main organic phase of bone extracellular matrix (ECM), is a promising candidate for printing bone scaffolds that mimic ECM, and several strategies have recently been explored to improve its printability, which is severely hampered by its poor visco‐elastic properties and slow gelation mechanisms.^11^, ^18^

Considering the soft nature and low viscosity of collagen, most of the resulting 3D printed scaffolds are characterized by low precision and simple architectures,^19^, ^20^ where its processability is usually increased mainly by exploiting mixing with other synthetic and natural polymers,^21^ or the use of low temperatures to combine cryogenic plotting and freeze‐drying.^22^ More recently, the production of more complex collagen‐based constructs has been explored, exploiting extrusion in printing baths capable of supporting the deposition of the soft biopolymer.^6^, ^18^

With the aim of improving the stability and mechanical stiffness of scaffolds, several studies have also reported the combination of collagen with inorganic phases acting as reinforcing agents, as well as the use of different strategies for chemical crosslinking.^11^, ^23^, ^24^, ^25^

Based on the natural composition of bone, hydroxyapatite (HA) particles or mesoporous bioactive glasses (MBGs) can be selected to create biomimetic composites as potential inorganic constituents.^26^, ^27^ Besides HA, which represents the main inorganic phase of bone ECM, MBGs have been widely recognized as effective osteoproductive materials due to their ability to promote HA crystals formation in the presence of physiological fluids.^28^, ^29^, ^30^

Despite the great advantages of using hybrid formulations combining polymeric and inorganic phases, their processability with 3D printing technologies is still critical, where high concentrations of inorganic phase and the further material aggregation can hinder proper extrusion, especially when using small diameter needles. Therefore, the design of scaffolds based on collagen‐based composite biomaterials and characterized by complex geometries requires adequate optimization of the overall printing process and is still a challenge to be addressed.

Taking in considerations all these aspects, and unlike what reported in the literature so far, the present work aimed at optimizing a material and manufacturing process suitable for the design of biomimetic scaffolds from both a chemical and structural point of view.

Based on previous studies by the authors,^30^, ^31^, ^32^ the combination of type I collagen with HA and MBG nanoparticles was explored to develop a hybrid formulation suitable for the extrusion printing of high precision biomimetic scaffolds intended for bone tissue regeneration. Indeed, the work is part of a larger study framed within the European project BOOST, where it was demonstrated how collagen‐based materials containing different bioactive inorganic phases are able to support growth and differentiation of human bone cells.^32^

As a first objective, a comprehensive physico‐chemical and rheological assessment was performed to explore the visco‐elastic properties of the collagen‐based biomaterial ink and the system's ability to reconstitute into a solid fibrous matrix once exposed to a physiologic temperature of 37°C. Based on rheological analysis, models based on computational‐fluid‐dynamics were implemented to describe the material flow regime and guide the subsequent optimization of process parameters. Furthermore, the prediction of the shear stress distribution enabled preliminary considerations to be made regarding the potential use of the developed formulation as a bioink.

In a second phase of the study, 3D scaffolds were produced by extrusion printing of the multiphase biomaterial ink using a gelatin‐based support bath, and according to the printing parameters previously predicted by the CFD model. Stabilization of the scaffolds after printing was achieved by incubation at 37°C and subsequent chemical crosslinking with genipin.^33^

The strategy developed and the careful investigation of material properties have enabled the successful design of collagen‐based biomimetic constructs with various geometries and high shape fidelity, with great potential in the field of bone tissue regeneration.

In addition to the biochemical stimulus, the opportunity to have scaffolds that faithfully reproduce the structural characteristics of bone tissue can further support cell differentiation and regeneration of physiologically healthy tissues.

## 2 MATERIALS AND METHODS

### 2.1 Preparation of the collagen‐based hybrid formulation (Coll/nanoMBG/nanoHA)

Starting from the volumetric ratio between organic and inorganic phases in natural bone (53% vol. of collagen and 47% vol. of inorganic phase)^34^ and considering a ratio of 50:50 between nanohydroxyapatite (nanoHA) and nanosized mesoporous bioactive glass (nanoMBG) particles, the final weight percentages of 45 wt% type I collagen powders, 40 wt% nanoHA and 15 wt% nanoMBG were calculated to prepare the collagen‐based formulation.

In details, nanoHA and nanoMBG were synthetized following protocols previously developed by the authors^31^, ^35^ and reported in the Data S1.

To obtain the final suspension, collagen powders (Blafar Ltd., Dublin, Ireland) were firstly dissolved in 0.5 M acetic acid at 4°C overnight. Parallelly, nanoHA rods were resuspended in a mixture of 1 M NaOH and Darvan 821‐A (0.5% v/v of the final volume) by sonicating for 1 h and stirring overnight to promote the proper dispersion of the inorganic phases. Particles of nanoMBG were resuspended in 0.5 M acetic acid, sonicated for 1 h and stirred for 2 h prior to mixing with the collagen solution. After the addition of the nanoMBG suspension, the mixture was stirred for about 1 h to ensure optimal homogeneity. Subsequently, the nanoHA suspension was added to the collagen/nanoMBG mixture dropwise, almost leading to pH neutralization. The pH of the suspension was further adjusted to 7.4 adding few drops of 1 M NaOH, reaching a final collagen concentration of 1.5 wt%. The resulting final suspension (Coll/nanoMBG/nanoHA) was stored at 4°C until used.

### 2.2 Rheological assessment

#### 2.2.1 Rheological tests

The rheological properties of the multiphase collagen‐based material were studied using a DHR‐2 controlled stress rotational Rheometer (TA Instruments, Waters, USA), while data collection and analysis was performed using the specialized software TRIOS (v5.1.1). For the experiments, the instrument was equipped with a parallel plate geometry with a diameter of 20 mm and a Peltier plate system to constantly control the temperature.

The variation in the viscosity of the suspension was measured by performing a flow ramp test at 10°C (printing temperature), varying shear rate values between 0.01 and 1000 s^−1^. These values were chosen to cover the shear‐rate range usually involved in extrusion 3D printing.^36^ A peak hold test at 10°C was carried out to simulate the printing process and investigate the shear recovery of the suspension, setting indicative shear stress values of 100 s^−1^ (100 s) and 0.1 s^−1^ (300 s) to reproduce the extrusion and deposition phases, respectively.

Yield point and flow point values were obtained from a dynamic amplitude sweep test performed on the suspension at 10°C, varying the oscillation stress between 0.1 and 100 Pa at a constant frequency of 1 Hz. Finally, the sol–gel transition of the hybrid formulation at 37°C was studied by means of time sweep tests maintaining a constant strain of 1% and a frequency of 1 Hz.

#### 2.2.2 Mathematical model

To estimate the printing parameters in advance, a CFD simulation was set up using COMSOL Multiphysics 6.0 (COMSOL Inc., Sweden). For this, the mathematical description of the flow behavior of the continuous phase (collagen solution) and of the mixture as a whole (Coll/nanoMBG/nanoHA) was needed. Therefore, the flow curves (viscosity vs. shear rate) were interpolated via the built‐in tool of the TRIOS software, following the Carreau‐Yasuda flow model^37^:

(1) $$\eta =\left(\eta_{0}-\eta_{\infty }\right)\left({\left(1+{\left(k\dot{\gamma }\right)}^{a}\right)}^{\frac{n-1}{a}}\right)+\eta_{\infty },$$

where η is viscosity, $\eta_{0}$ and $\eta_{\infty }$ are respectively the zero‐shear and infinite‐shear viscosities, k the consistency (characteristic time), $\dot{\gamma }$ is the shear rate, n the power law index and a a parameter describing the transition between Newtonian plateau and power law region. The obtained values of the model parameters were used to describe the flow properties of the collagen solution and the Coll/nanoMBG/nanoHA system in COMSOL.

### 2.3 CFD model

The computer‐aided design (CAD) of the fluid (Figure S1) was obtained as the volume enclosed by the geometry of the printing cartridge (Cellink, 3 ml) and needle (Cellink, G27) (whose CAD models were kindly provided by Nordson, U.S.A.) via the SolidWorks software (ver 2019–2020, Dassault Systemes, France). The fluid geometry was imported into COMSOL and treated as an axial symmetric solid to speed up the computation. The flow behavior of the suspension was simulated via the Laminar Flow and Phase Transport physics coupled by a Mixture Model Multiphysics node. The flow properties of the collagen phase and of the suspension were implemented according to the Carreau‐Yasuda fitting from Section 2.2.2. Instead, the density values used were 0.45, 0.9 and 3.1 g/cm^3^ for, respectively, collagen, nanoMBG and nanoHA. Both nanoMBG and nanoHA were modeled as solid spherical particles with radius and concentration of 100 nm and 15% for nanoMBG and 20 nm and 40% for nanoHA. The imposed boundary conditions were a pressure at the inlet varying from 30 to 50 kPa, no‐slip condition at all walls and atmospheric pressure at the extrusion needle outlet. The model was meshed with free triangular elements automatically calibrated for fluid dynamics, with an extremely fine density. Additionally, two boundary layers of quadrilateral elements were introduced to resolve the thin boundary layers along the no‐slip boundaries. The mesh resulted in 16,247 triangles and 1866 quads for a total of 18,113 elements and 54,595 degrees of freedom. A stationary solver was used to simulate steady‐state flow conditions.

### 2.4 Fabrication of scaffolds via extrusion printing

#### 2.4.1 Preparation of the gelatin‐based supporting bath

To support the deposition of the material filaments and prevent the structure from collapsing during printing, a gelatin slurry was obtained from LifeSupport dried powders (FluidForm, Acton MA, USA) consisting of gelatin microparticles of defined size and shape, following the supplier's instructions for preparation.^18^

In details, 1 g of LifeSupport dried powders were rehydrated in 35 ml of prechilled Dulbecco Phosphate Buffered Saline (D‐PBS, Sigma Aldrich, Milan, Italy) for 10 min using 50 ml falcon tubes. The suspension was centrifuged at 600 rcf for 5 min to compact the particles at the bottom of the tube enabling the removal of the supernatant. The tube was shaken for few seconds until the hydrated particles started flowing and subsequently centrifuged again at 1000 rcf, for 5 min. After aspiration of the residual supernatant, the resulting slurry was poured into the desired container (2‐wells chamber slides), avoiding the introduction of air bubbles.

The volume of slurry poured in the container was adjusted according to the dimensions of the 3D model, always resulting in a supporting bath at least 2 mm thicker than the desired thickness of the scaffold.

#### 2.4.2 Printing tests

All the printing tests were performed using a temperature controlled pneumatic printhead mounted on a BIOX 3D Bioprinter (BIOX, Cellink, Sweden) and 27 G metal needles (inner diameter of about 120 μm). The print‐head temperature was set at 10°C while the print‐bed was kept at 20°C to maintain the viscoelastic properties of the biomaterial ink and the gelatin‐bath constant throughout the process.

A 3D digital model for square scaffold (10 × 10 × 1 mm) was selected from the .STL files library provided by the bioprinter manufacturer. A grid pattern with a filling density of 15% was configured to obtain 3D structures with a pore size of 2.2 mm, to allow a simple analysis of the resulting printing fidelity. After optimization of the printing parameters and as proof of concept, different models (e.g., honeycomb and gyroids) were selected to obtain alternative scaffold morphologies with high shape fidelity.

Based on the results obtained from the computational model, the final optimization of the extrusion parameters was obtained setting a layer height of 170 μm (about 60% of internal diameter), varying print‐head speed (2–7 mm/s) and air pressure (30–50 kPa), according to the resulting accuracy of the filament extruded. Both the printing settings and the 3D model features were configured using the integrated software (heartos_v1.7.0 + 165‐dev) of the bioprinter.

Due to the large number of tests conducted, only a few images of the scaffolds will be shown in the Results section, with the aim of discriminating the contribution made by pressure and speed variations.

#### 2.4.3 Post‐processing and chemical crosslinking

After printing, 3D scaffolds were incubated overnight at 37°C in to promote the sol–gel transition of the collagen‐based material, subsequently melting and removing the gelatin slurry. To remove any gelatin residue, the scaffolds were washed twice in dH_2_O under gentle agitation (50 rpm).

To increase mechanical and thermal stability, the 3D scaffolds were chemically crosslinked using 0.5 wt% genipin (Challenge Bioproducts, Taiwan) in 70% ethanol solution (GEN/EtOH), following a procedure previously optimized by the authors.^33^ Briefly, 3D scaffolds were immersed in 3 ml of GEN/EtOH at 37°C for 24 h to promote the covalent crosslinking of collagen. The samples were then washed twice in dH_2_O to remove residual crosslinker.

#### 2.4.4 Assessment of printing fidelity

The 3D constructs were visually inspected and imaged immediately after removal of the supporting gelatin slurry, and after chemical crosslinking in order to assess the shape fidelity on the x, y and z axes.

The images obtained were analyzed by means of the ImageJ software, where scaffold measurements were collected for the geometry sides and for individual strands. Five measurements were chosen for each scaffold (Figure S3A), and the final value presented as mean and standard deviation.

### 2.5 Scaffold characterization

#### 2.5.1 Physico‐chemical and morphological assessment

Morphological evaluation was performed to observe the reconstitution of collagen fibrils at physiological temperature and pH, and the distribution of the inorganic phases into the collagen matrix.

Before printing, bulk samples of Coll/nanoMBG/nanoHA were obtained by pipetting the collagen‐based suspension in a silicon mold, that was subsequently incubated at 37°C for 3 h.

For the analyses, both bulk samples and chemically crosslinked 3D printed scaffolds were lyophilized for 24 h after freezing at −20°C, using a Lyovapor L‐200 freeze‐dryer (Büchi, Switzerland) under vacuum (<0.1 mbar). Cross‐sections of lyophilized samples were sputter‐coated with platinum (7 nm thickness layer) and analyzed through Field‐Emission Scanning Electron Microscopy (FESEM) with a ZEISS MERLIN instrument (Carl Zeiss AG, Oberkochen, Germany).

The chemical composition of the developed biomaterial was then confirmed by means of Fourier‐transform infrared spectroscopy (FTIR) and by using the attenuated total reflection (ATR) mode. The resulting spectrum, in the 4000–650 cm^−1^ range, were collected by using a Bruker Equinox 55 spectrometer, equipped with MCT cryodetector, at a spectral resolution of 4 cm^−1^ and accumulation of 32 scans.

#### 2.5.2 Strength and thermal stability

Rheological analyses were performed to investigate the change in the visco‐elastic properties of the multiphase material before and after the chemical treatment with genipin, in order to demonstrate the effective crosslinking of collagen, i.e., the increase in material stiffness and temperature stability. All the tests were performed using the rotational rheometer and the same experimental set‐up reported in Section 2.2.1.

In detail, a dynamic amplitude sweep was carried out on the samples before and after chemical crosslinking by varying the strain rate between 0.01% and 1% at a constant frequency of 1 Hz and at 37°C, measuring the value of storage (*G*′) and loss (*G*″) moduli. The same samples were subjected to an oscillation temperature ramp between 25°C and 80°C under 1% strain and 1 Hz with a ramp rate of 5°C/min, to detect the denaturation temperature of the material associated with the sharp decrease in the overall complex modulus (*G*′, *G*″).

## 3 RESULTS AND DISCUSSION

### 3.1 Preparation of the Coll/nanoMBG/nanoHA system

Based on the composite nature of bone tissue, type I collagen, nano‐sized mesoporous bioactive glasses (nanoMBGs) and hydroxyapatite nanorods (nanoHA) were combined to develop a bioactive collagen‐based biomaterial ink suitable for extrusion printing technologies. Type I collagen and hydroxyapatite represent the main organic and inorganic phase of bone tissue respectively, while MBGs are well known for their high bioactive and pro‐osteogenic characteristics.^35^, ^38^ Consequently, the synergistic effect of these materials can be exploited to design biomimetic and bioactive systems with great potential for bone tissue regeneration.^28^, ^39^, ^40^

Spherical nanoMBG with a size between 100 and 500 nm were produced following the optimized sol–gel route (Figure S2A) reported by the authors in a previous work,^35^ where the resulting particles had a surface area between 400 and 500 m^2^/g, and mesopores of 2–4 nm. Based on another study by the authors,^31^ a hydrothermal method, combined with the use of the cytocompatible dispersing agent Darvan 821‐A, was optimized to obtain uniform‐sized rods with length of 40–60 nm and a width of 20 nm (Figure S2B), thus mimicking the morphology and the size of HA crystals present in bones (50 × 25 × 2 nm).

After preparation, nanoMBG and nanoHA were combined with type I collagen respecting the volume ratio between organic and inorganic phases in the natural bone (53% vol. of collagen and 47% vol. of inorganic phase).^34^ To reduce the presence of inorganic phase aggregates, homogeneous suspensions of nanoMBG and nanoHA were obtained prior to addition to the collagen solution. Mixing the different phases and adjusting the pH of the suspension to 7.4 were carried out at 10°C to prevent premature gelation of the system and better preserve the physico‐chemical properties of the protein.

The resulting Coll/nanoMBG/nanoHA suspension proved its stability at temperatures lower than 10°C without evidencing phase separation or particle sedimentation.

The sol–gel transition of the multiphase system triggered by a physiological temperature of 37°C was demonstrated by formation of bulk samples, that were subsequently frozen and freeze‐dried to perform the morphological evaluation.

**FIGURE 1:**
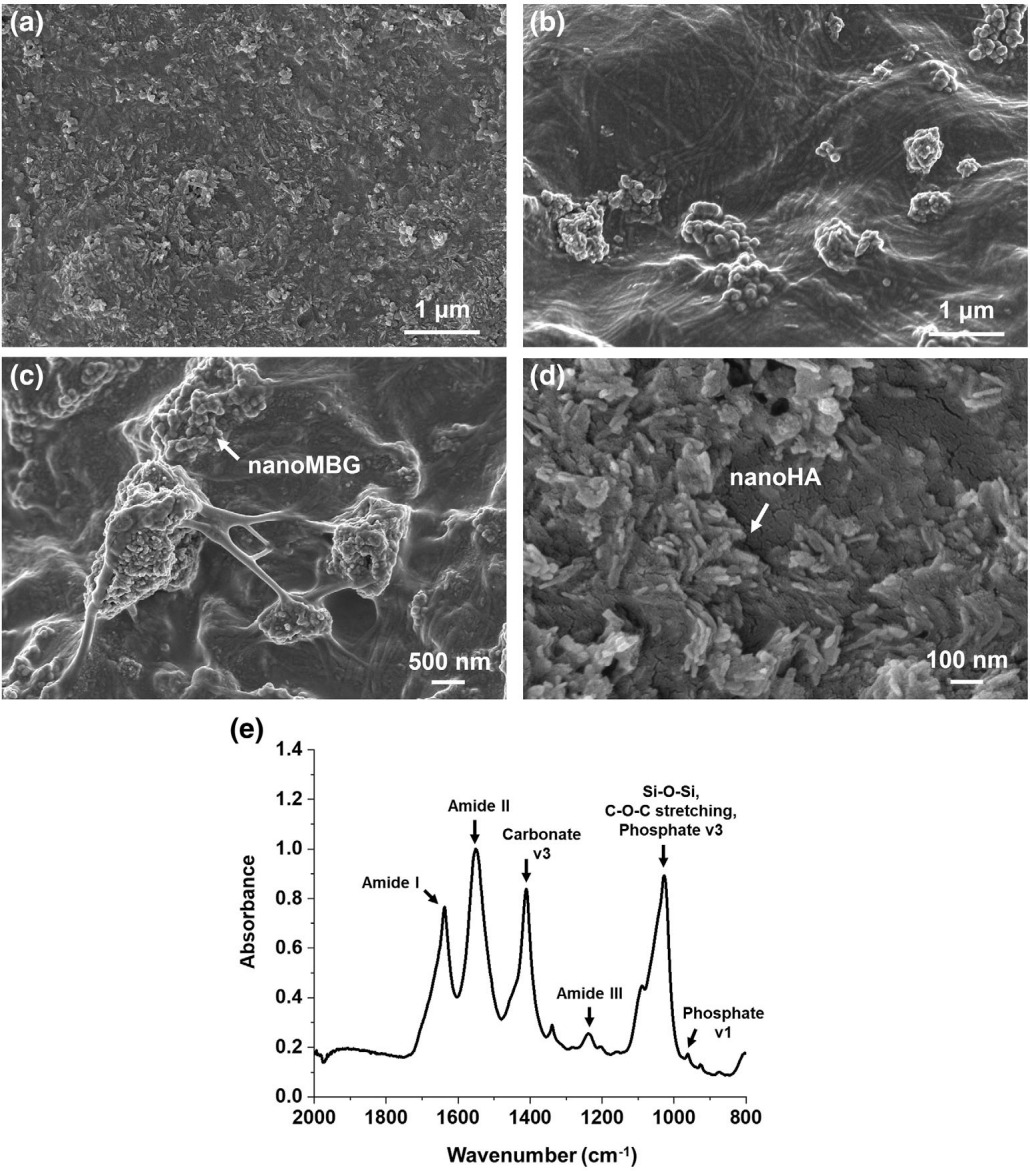
Cross‐sectional FE‐SEM images at different magnifications (a–d) and ATR‐FTIR spectrum (e) of Coll/nanoMBG/nanoHA samples. The white arrows indicate the different inorganic phases embedded into the collagen matrix

As shown in Figure 1, FE‐SEM images of sample cross‐section proved the reconstitution of a solid matrix composed of collagen fibrils (Figure 1a,b), as well as the good distribution and incorporation of rod‐like nanoHA (Figure 1d) and spherical nanoMBG particles (Figure 1c), highlighting only the presence of a few aggregates lower than 5 microns in size. The good dispersion of the inorganic phases into the collagen matrix was considered of particular interest and importance in view of the proper extrudability of the developed biomaterial ink.

The ATR_FTIR analysis confirmed the multiphase nature of the developed Coll/nanoMBG/nanoHA system. The resulting spectrum, represented in Figure 1e, clearly shows the typical peaks of collagen centered at 1637 cm^−1^ for amide I (C=O stretching), at 1551 cm^−1^ for amide II (N—H stretching) and at 1238 cm^−1^ for amide III (C—N bending). In parallel, the presence of HA was confirmed by the phosphate peaks centered at 1089 cm^−1^ and 963 cm^−1^, while the peak at 1027 cm^−1^ resulted from the contribution of both Si—O—Si groups of nanoMBG particles and the phosphate of nanoHA.^28^, ^41^

### 3.2 Rheology of the multiphase system

Although the development of multiphase systems provides the opportunity to combine the positive effects of multiple materials to mimic the native composition of the tissue, their processing with 3D printing technologies requires a comprehensive and accurate evaluation to define the most promising manufacturing method.

Consequently, the rheology of the developed formulation was explored to assess its potential printability and the most suitable post‐processing strategies, by measuring the visco‐elastic properties of the collagen‐based material at 10°C and upon reconstitution of the solid fibrous matrix at 37°C.

**FIGURE 2:**
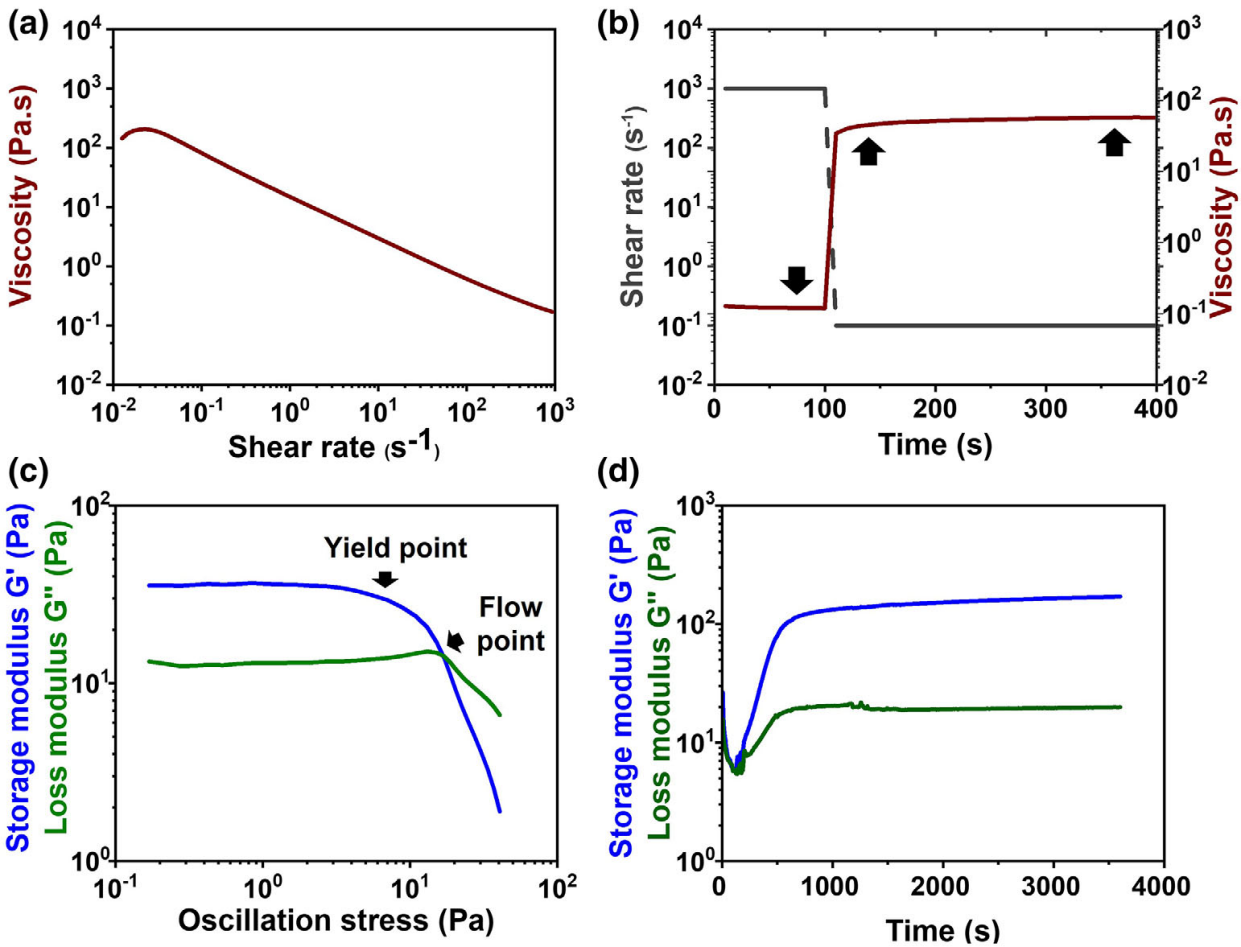
Rheological properties of the Coll/nanoMBG/nanoHA system: Shear‐thinning (a), shear‐recovery (b), yield and flow points (c) measured at 10°C and sol–gel transition of the material at 37°C (d) [Color figure can be viewed at wileyonlinelibrary.com]

Figure 2a shows the pseudo‐plastic behavior (shear thinning) of the multiphasic system when subjected to increasing values of shear rate (0.01–1000 s^−1^) at 10°C, evidenced by the decrease of viscosity values from about 206.7 to 0.17 Pa s. This is particularly favorable in the case of materials intended for 3D printing applications, as it allows for proper extrusion especially with thin needles involving high shear rates and stresses during the process.

Besides shear thinning, rapid shear recovery is normally required to preserve size and shape of deposited strands, enabling good printing fidelity.^7^, ^42^ Based on these observations, the suspension shear recovery was studied by observing the variation in material viscosity subjected to zero‐shear conditions immediately after the removal of a high applied shear rate of 1000 s^−1^. As shown in Figure 2b, the analysis evidenced a slow increase and not a complete recovery of viscosity even 300 s after the shear stress was removed. Moreover, yield point and flow point of the system were measured by means of the amplitude sweep, where the complex modulus of the material is represented as a function of shear stress at 10°C (Figure 2c). The yield point of about 3.6 Pa was defined as the limit of the linear viscoelastic range, while the flow point was measured to be of about 16.4 Pa, where the loss modulus *G*″ is above the elastic modulus *G*′ and therefore flow can occur.^8^ In this case, the detection of low values of yield and flow points, as well as the low shear recovery, highlighted the need to optimize strategies to ensure the correct support of the material after extrusion.

To complete the rheological assessment of the multiphase formulation, the sol–gel transition triggered by the ordered packing of collagen molecules forming 3D fibril structures was studied by means of a time sweep test (Figure 2d), in which the suspension was loaded at 10°C and immediately subjected at a constant temperature of 37°C and oscillatory stress (1% strain, 1 Hz). After 1 h, the storage modulus of the system visibly increased denoting the creation of a more stable and solid gel, with values of 170.3 Pa and 19.9 Pa for *G*′ and *G*″ values, respectively. Although gel formation was successful after approximately 500 s of exposure a 37°C, the time required for collagen to self‐assemble further suggested the need for a supporting material to achieve adequate printing fidelity.

### 3.3 Study of fluid dynamics

**FIGURE 3:**
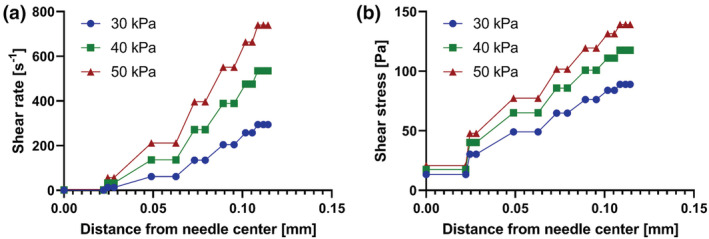
Shear rate (a) and shear stress (b) values at different distance from needle center calculated by CFD model and in relation to different printing pressures [Color figure can be viewed at wileyonlinelibrary.com]

As previously mentioned, the suspension exhibited pseudo‐plastic trend in the shear‐rate range of measurement. This is further supported by the Carreau‐Yasuda fitting, whose parameter values can be seen in Table 1, and by the parameter *k* in particular. In fact, the inverse of *k* gives the shear rate at which a fluid behavior shifts from Newtonian to pseudo‐plastic.^43^ In this case, this value equals to 0.03 s^−1^, well below the usual shear rates in extrusion printing (Figure 3a).

**TABLE 1:** Carreau‐Yasuda parameters for collagen solution and Coll/nanoMBG/nanoHA, at 10°C, used to implement the CFD model

System	$\eta_{0}$ [Pa s]	$\eta_{\infty }$ (Pa s)	k (s)	n	a	$R^{2}$
Coll solution	58.2	0.07	35.0	0.09	76.9	0.99
Coll/nanoMBG/nanoHA	169.3	0.07	28.7	0.27	69.6	0.99

Because of the above considerations, conventional analytical models to estimate the volume flow rate (and consequently the printhead translational speed), such as that introduced by Khalil and Sun,^44^ are not applicable, being valid only in the case of Newtonian fluids. Therefore, we run CFD simulations to properly evaluate the suspension flow behavior in our printing system and to estimate the printhead speed in advance.

Applying as inlet pressure the values 30, 40 and 50 kPa, CFD simulations calculated printhead speed values of 1.9, 3.7 and 6.0 mm/s, respectively. These were then used as starting reference to optimize the printing procedure, as discussed in the following section. Additionally, the analysis allowed to quantify the shear rates and stresses within the needle in order to make some preliminary considerations about the potential use of the developed formulation as bioink for future applications. As can be seen from Figure 3, the shear‐rate and shear‐stress increases from the needle center to the wall, a behavior typical of flow in cylindrical pipes,^45^ and as a function of applied pressure for a given distance from the needle center. According to the study conducted by Han and coworkers^46^ and related to the process‐induced cell damage in extrusion bioprinting, needle diameter and length, as well as the operating pressure, determinate shear stress distribution, ultimately influencing cell survival. In this frame, they observed significant cell damage for pressures exceeding 40 kPa and for increasing values of needle length (>11 mm) and diameter (>200 μm), associated with shear stresses higher than 250 Pa. As visible in Figure 3b, the CFD analysis based on the rheological properties of Coll/nanoMBG/nanoHA, and the geometrical parameters of the extruder, led to the prediction of shear stress values lower than 200 Pa even for an operating pressure of 50 kPa, suggesting the potential suitability of the developed material as bioink.

### 3.4 Scaffolds fabrication via extrusion printing

The printability of the developed multiphase formulation was further assessed using an extrusion‐based system, equipped with 27 G needles and characterized by an inner diameter of about 200 μm, to potentially achieve a high resolution of the final 3D constructs.

**FIGURE 4:**
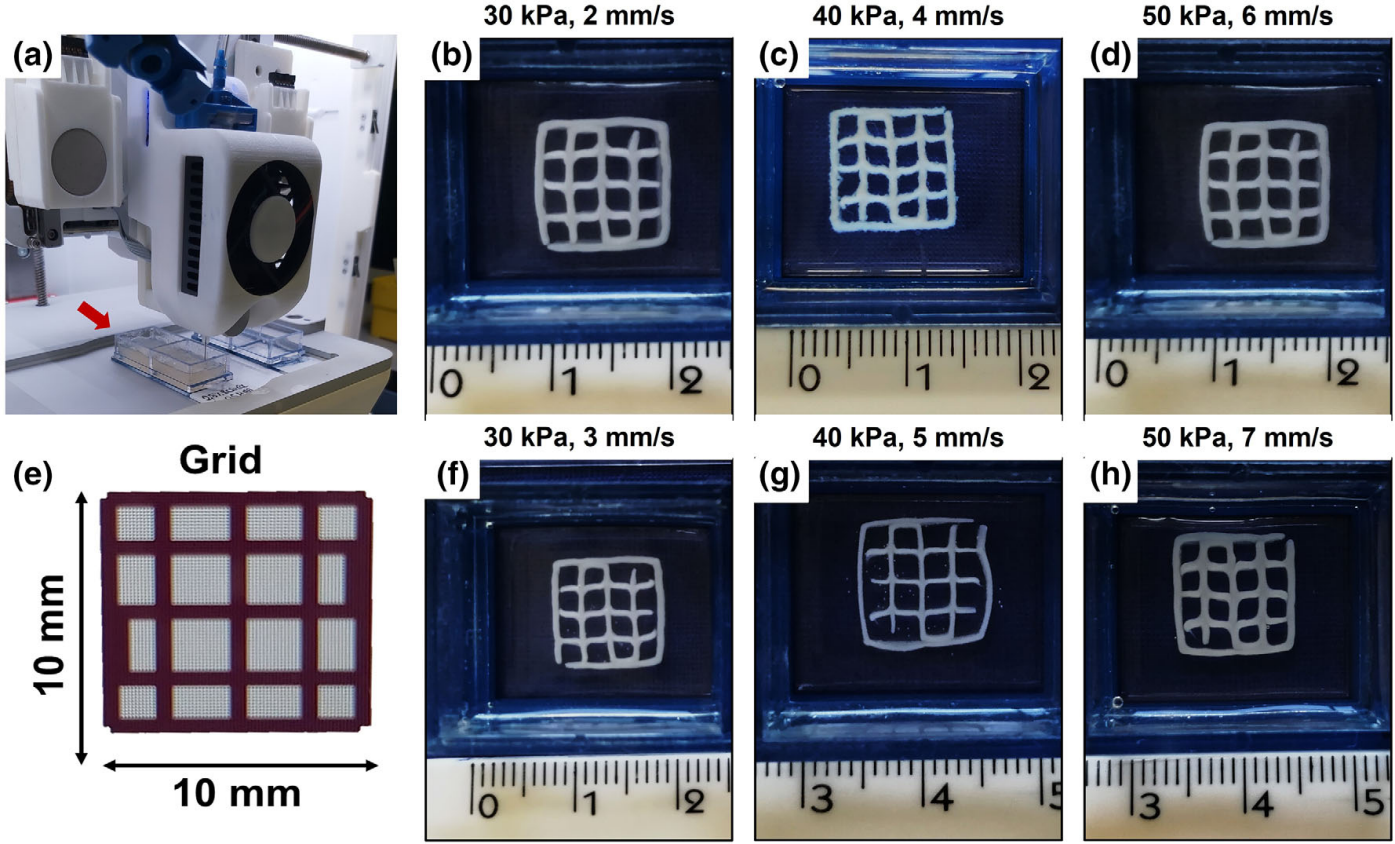
Images of the printing set‐up (a), scaffolds obtained for different printing parameters (b, c, d, f, g, h) and the corresponding CAD model (e) [Color figure can be viewed at wileyonlinelibrary.com]

In detail, a temperature controlled pneumatic printhead was used to enable the material extrusion driven by regulating the air‐pressure on the cartridge and keep a constant temperature of 10°C to preserve the visco‐elastic properties of the collagen‐based suspension. In parallel, as shown in Figure 4a, 2‐well chamber‐slide containers were filled with the freshly prepared supporting gelatin slurry and placed on the printing‐bed kept at 20°C to avoid the material melting.

For the experimental tests, a 3D digital model for square scaffolds (10 × 10 × 1 mm) with a grid pattern and infill density of 15% (Figure 4e) was selected to easily allow the evaluation of shape fidelity and the deposition of material strands.

Starting from the results of CFD simulations, pressures of 30, 40, and 50 kPa were combined with printhead speeds ranging between 2 and 7 mm/s, while the z step was set to 170 μm (about 60% of inner diameter) to favor the adhesion of subsequent layers while avoiding their collapse.^7^, ^8^

After printing, the resulting scaffolds were incubated at 37°C, overnight, to promote the sol–gel transition of the Coll/nanoMBG/nanoHA system, while removing the liquid gelatin. Subsequently, the resulting structures were visually inspected and measured to define the accuracy of the extruded filament and the printing parameters that provide the best shape fidelity (Figure 4b,c,d,f,g,h).

The experimental evaluation confirmed the processability of the Coll/nanoMBG/nanoHA formulation with extrusion‐based systems, where the shear‐thinning behavior of the material allowed for the formation of continuous flow of material, without highlighting needle clogging even using small extruders and thus suggesting good dispersion of the inorganic phases. Furthermore, during the printing process, the rheological behavior exhibited by the gelatin slurry at 20°C enabled the needle to move accurately due to the negligible mechanical resistance, keeping the deposited material in place.

The subsequent incubation at 37°C triggered the sol–gel transition of the material, allowing for the removal of the supporting gelatin slurry and avoiding residues between the pores of the printed structures.

At this stage, scaffolds were imaged and measured with the support of the ImageJ software, allowing the measurements of the printed constructs to be compared with those of the digital model (Figure 4e).

As indicated in the methodology section, printing tests were performed by modifying each parameter individually to ensure maximum comprehension of the results, but due to the large number of tests, only the most significant ones are reported.

The printability assessment started exploring the combination of pressure and printhead‐speed values predicted by the computational model (Figure 4b,c,d) over a pressure and print‐head speed range of 30–50 kPa and 2–6 mm/s, respectively. In accordance with the CFD analysis and the shear thinning behavior of the developed Coll/nanoMBG/nanoHA suspension, the increase in material flow caused by higher pressures was compensated by higher printhead speeds, successfully leading to the replication of the CAD geometry, with scaffolds presenting defined pores and strands. However, comparison with the CAD measurements (Table 2, Table S2) showed that the parameters predicted by CFD analysis led to the formation of filaments with larger size.

**TABLE 2:** Set of measurements obtained from ImageJ analyses for the CAD model and the produced scaffolds

Printing parameters	Scaffold sides (mm)				Scaffold strand (mm) mean ± SD
CAD model (Figure 4e)	9.44	10.16	9.47	10.03	0.56 ± 0.07
30 kPa; 2 mm/s (Figure 4b)	11.62	11.93	12.37	12.18	0.72 ± 0.05
30 kPa; 3 mm/s (Figure 4f)	11.39	11.64	11.82	11.14	0.54 ± 0.05
40 kPa; 4 mm/s (Figure 4c)	16.23	15.92	16.30	15.92	0.88 ± 0.09
40 kPa; 5 mm/s (Figure 4g)	11.77	11.55	12.27	11.94	0.43 ± 0.06
50 kPa; 6 mm/s (Figure 4d)	13.40	13.86	14.26	14.33	0.75 ± 0.03
50 kPa; 7 mm/s (Figure 4h)	9.75	10.35	10.50	10.55	0.54 ± 0.09

With the aim to increase the final shape fidelity, the printhead speed was increased up to 3, 5 and 7 mm/s according to the set pressure (30, 40 and 50 kPa). As reported in Figure 4 (Figure 4f,g,h) and Table 2, a reduction of approximately 20% in the diameter of the filament was achieved by setting speeds of 3 and 7 mm/s combined with pressures of 30 kPa and 50 kPa respectively, finally achieving congruence between the measurements of the scaffold and CAD model. A similar result was obtained by setting 40 kPa and 5 mm/s as printing pressure and speed, where the reduction in strand diameter was up to approximately 45%.

When comparing the printhead speed predicted by the computational simulation and the actual values used, discrepancies could be seen. These can be ascribed to several factors, firstly the shape of nanoHA particles. The COMSOL software allows to model the particles of the dispersed phase only as spheres, whereas nanoHA particles are in the shape of cylinders. Even by using the approach of the volume‐equivalent sphere,^45^ spherical particles would not exhibit a preferred orientation along the flow because of their isotropy. Instead, cylindrical particles would have a preferred orientation under flow conditions, depending on their height‐to‐radius ratio. This might affect how streamlines would converge at the entrance of the needle, and consequently influence the final flow rate. However, it is important to note that the data from the CFD simulation were used just as initial guideline for the printing process and was in any case the result of a simplification, which also allowed for a significant reduction in the number of tests. Based on the experimental assessment, 30 kPa and 3 mm/s were selected as the best parameters to achieve 3D structures accurately reproducing the characteristics of the CAD model, without showing evident deformations. Furthermore, as evidenced by the CFD analysis (Figure 3) and in view of a potential future application in bioprinting, lower pressures are normally preferred to prevent cell damage caused by high shear stresses within the needle.^46^

### 3.5 Chemical crosslinking of Coll/nanoMBG/nanoHA scaffolds

After optimization of the printing parameters, the scaffolds obtained by extrusion printing of the developed Coll/nanoMBG/nanoHA suspension were crosslinked by means of a 0.5 wt% genipin in 70% ethanol solution (GEN/EtOH), to promote the formation of covalent bonds between collagen molecules and the consequent increase in material stability. According to the literature,^19^, ^47^ genipin is a naturally‐derived cytocompatible agent that causes the crosslinking of free amino groups of collagen through the formation of cyclic structures, which act as intra‐ and inter‐molecular bridges along the fibers.

Following a previous protocol developed by the authors,^33^ the scaffolds were immersed in GEN/EtOH solution, promoting chemical crosslinking at 37°C for 24 h, and then washed to remove any residual genipin.

The effects of the chemical treatment on the final resolution and definition of the 3D mesh‐like constructs were explored by visual inspection and by comparing the final dimensions with those of non‐crosslinked scaffolds (Figure S3, Table S2).

**FIGURE 5:**
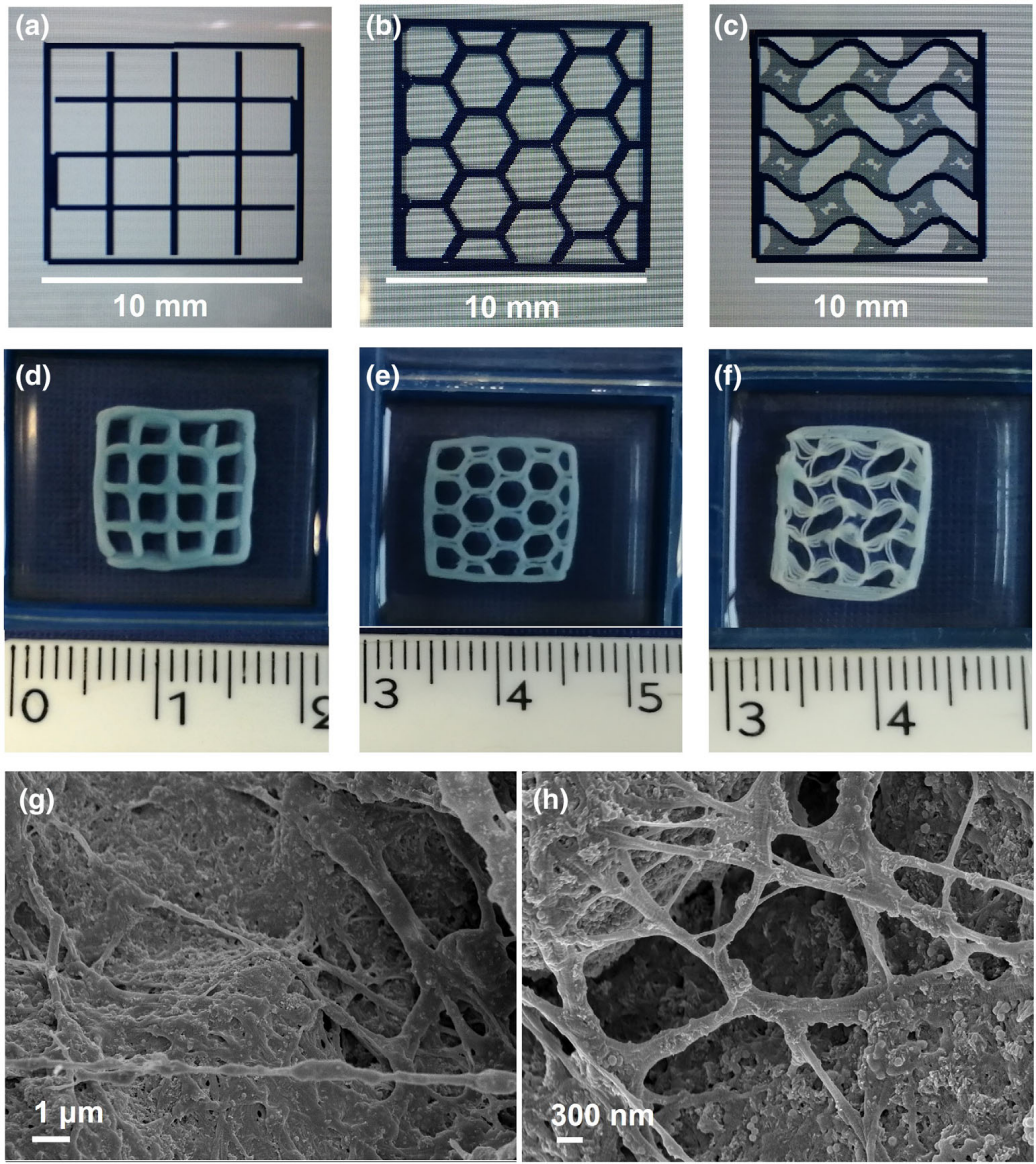
Images of CAD models (a, b, c) and the respective chemically crosslinked 3D printed structures (d, e, f). FE‐SEM images of mesh‐like scaffold cross‐section at different magnifications showing inorganic phases embedded into the collagen fibrillar matrix (g, h). White scale bars in panels (a, b, c) are equal to 10 mm. [Color figure can be viewed at wileyonlinelibrary.com]

Alongside the macro‐scale properties of the final constructs, morphological analyses were conducted to observe the micro and nano‐architecture of the material, resulting from the printing process and chemical crosslinking treatment (Figure 5g,h).

As shown in Figure 5 (Figure 5a,d) and S3 (Figure S3A,B), the genipin‐treated scaffolds retained the morphological features of the non‐crosslinked printed structures, without showing significant changes in filament dimensions (Table S2). The crosslinking treatment also led to the overall increase in scaffold stiffness, allowing easier handling and preservation of its thickness (Figure S3C).

To further demonstrate the potential of the developed method in the creation of scaffolds with high shape fidelity, Coll/nanoMBG/nanoHA ink was used to print 3D complex constructs characterized by honeycomb and gyroid morphologies, as represented by the CAD models in Figure 5b,c. The scaffolds were printed using the previously optimized parameters (30 kPa; 3 mm/s), incubated at 37°C and further chemically crosslinked with genipin.

As can be seen in Figure 5e,f, the optimized methodology proved to be successful in the design of scaffolds with different geometries, ensuring that the morphological characteristics established by the CAD model were preserved.

Moreover, the morphological evaluation by FE‐SEM (Figure 5g,h) confirmed the successful self‐assembly of collagen and the subsequent formation of a highly fibrillar structure for the crosslinked scaffolds, as found in the previous analysis on bulk samples (Figure 1a–d). These observations thus suggested that the overall printing steps do not to alter the homogeneous distribution of inorganic phases in the collagen matrix, where FE‐SEM images also showed the distribution of nanoMBG and nanoHA particles alongside collagen fibers.

**FIGURE 6:**
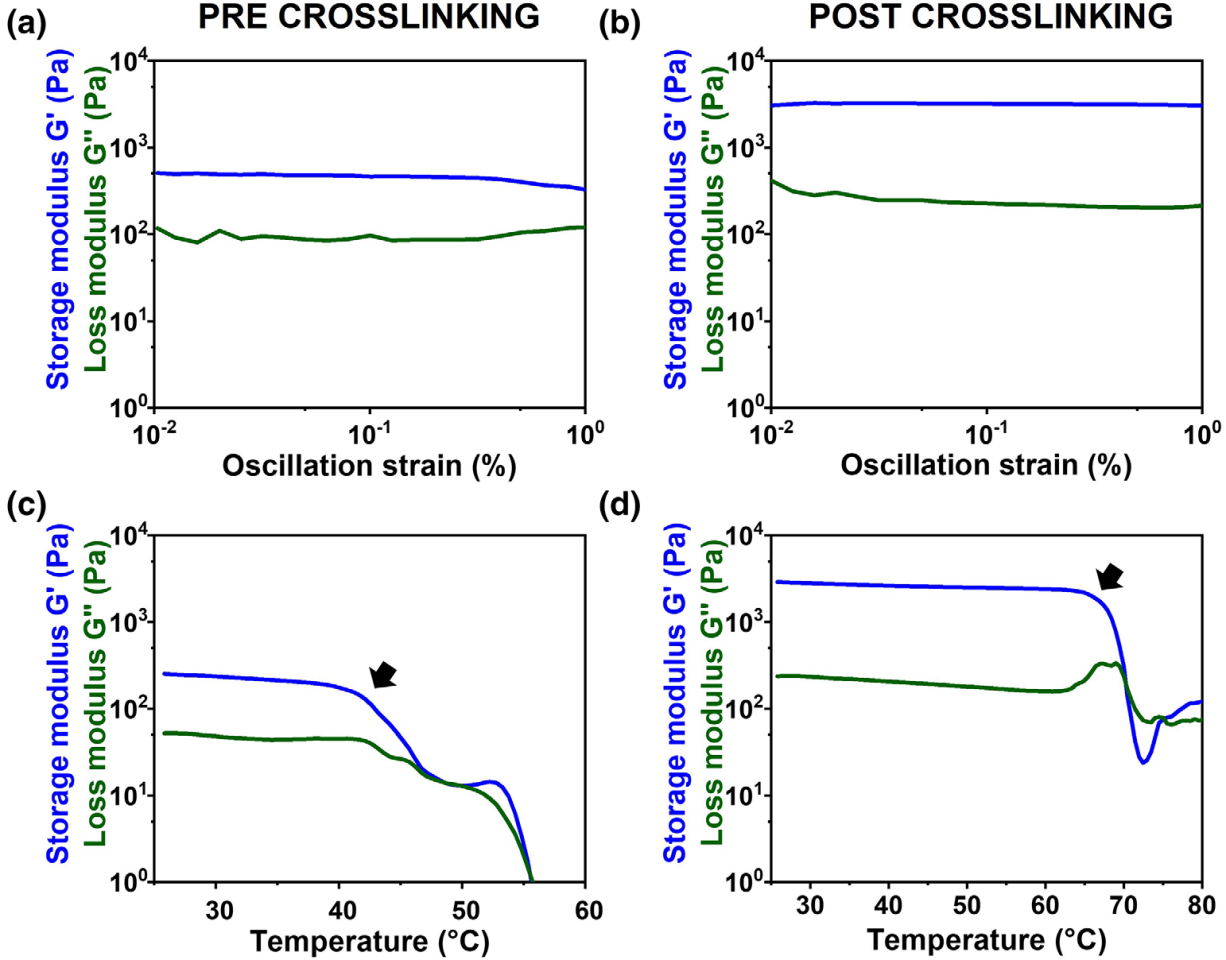
Amplitude sweep tests (a, b) and temperature ramp (c, d) performed on Coll/nanoMBG/nanoHA before and after chemical crosslinking with GEN/EtOH [Color figure can be viewed at wileyonlinelibrary.com]

To explore the reinforcing effect caused by the formation of covalent bonds between collagen molecules, the visco‐elastic properties of the Coll/nanoMBG/nanoHA system before and after the treatment with genipin were studied by means of rheological tests (Figure 6). The increase in material strength was investigated by means of a dynamic amplitude sweep test (0.01%–1% strain; 1 Hz), while a temperature ramp was performed to emphasize the thermal stability of the system.

As shown in Figure 6 (Figure 6a,b) and Table S3, the treatment with genipin caused the increase in *G*′ and *G*″ values from about 369 Pa up to 3100 Pa. In parallel, the temperature ramp evidenced a significant shift in the denaturation temperature of collagen, where the collapse of the material structure occurred at 42°C and 65°C for the non‐crosslinked and crosslinked Coll/nanoMBG/nanoHA system respectively (Figure 6c,d). Effective crosslinking was thus demonstrated by an almost tenfold increase in the system's storage modulus and denaturation temperature.

The overall results proved the successful design of 3D‐printed biomimetic constructs characterized by high shape fidelity and mechanical and thermal stability due to the effective crosslinking of collagen. The comprehensive rheological assessment and the computational study performed on the developed collagen‐based formulation enabled the most suitable extrusion printing process to be set up, highlighting the great potential of the multiphase system as a biomaterial ink.

These results lay the basis for the realization of biomimetic scaffolds capable of mimicking with good accuracy not only the composition but also the architecture of the tissue to be regenerated. In this context, a comprehensive biological study will be conducted in the presence of human osteoblasts and osteoclasts cocultures, to determine the effects of the biochemical and structural stimulus on cell response.

Moreover, based on the preliminary results obtained from the CFD model, future experiments will explore the potential use of the developed Coll/nanoMBG/nanoHA system as a bioink, to enable the effective bioprinting of scaffolds and design more reliable in vitro bone models.

## 4 CONCLUSION

In conclusion, a multiphase biomaterial ink suitable for extrusion printing was developed, that combines type I collagen with nanosized hydroxyapatite and mesoporous bioactive glass particles, with the aim of mimicking the natural composition of bone tissue. Rheological evaluation of the developed system demonstrated its shear‐thinning behavior and extrudability even with small diameter needles, which was further confirmed by the computational‐fluid‐dynamic model.

The low yield stress value and slow shear recovery of the system was compensated by the use of a supporting gelatin slurry during the printing process and the subsequent incubation at 37°C, to induce the sol–gel transition and thus the formation of collagen fibrils.

The definition of the printing process was based on the pressure and speed values predicted by the CFD analysis, where experimental tests performed with a temperature controlled pneumatic print‐head almost confirmed the results derived from the computational model. In particular, the slight increase of print‐head speed over a pressure range of 30–50 kPa led to the design of scaffolds with high printing fidelity, capable of faithfully reproducing CAD models with different geometries (grid, honeycomb, gyroid).

Upon optimization of the printing process and parameters, the mechanical and thermal stability of the 3D constructs was improved by chemical crosslinking with genipin, without altering the final shape fidelity.

Overall, these results proved the suitability of the developed multiphase system as a biomaterial ink, following the development of a printing strategy optimized on the measured rheological properties. Furthermore, the shear stress distribution within the needle predicted by the CFD analysis suggested the potential use of the material as a bioink.

Finally, the comprehensive rheological assessment and further methodology used to define the shear‐conditions and related optimal printing parameters may provide building blocks for other researchers to use the collagen‐based multiphase ink with the wide range of available 3D printing platforms.

## AUTHOR CONTRIBUTIONS

**Giorgia Montalbano:** Conceptualization (equal); data curation (lead); formal analysis (lead); investigation (equal); methodology (lead); software (equal); validation (equal); visualization (equal); writing – original draft (lead). **Andrea Roberto Calore:** Data curation (supporting); formal analysis (equal); methodology (supporting); software (equal); validation (equal); visualization (supporting); writing – original draft (supporting). **Chiara Vitale‐Brovarone:** Conceptualization (equal); funding acquisition (lead); investigation (equal); project administration (lead); resources (lead); supervision (lead); writing – review and editing (lead).

## Supporting information


**Data S1**. Supporting Information.

## Data Availability

The data that support the findings of this study are available from the corresponding author upon reasonable request.
