# Supplementary material for: Extrusion 3D printing of a multiphase collagen‐based material: An optimized strategy to obtain biomimetic scaffolds with high shape fidelity
Source: J Appl Polym Sci. 2023 Jan 9;140(10):e53593. doi: 10.1002/app.53593 (PMC10078475; doi:10.1002/app.53593)
Supplement: Supplementary file 1 — Data S1. Supporting Information. [file APP-140-0-s001.docx]

Supporting Information

**Supplementary material for Section 2.1**

**Protocol for the synthesis of rod-like nanoHA particles adapted from Montalbano et al.**^31^

5.48 g of potassium phosphate dibasic trihydrate were dissolved in 100 mL of double distilled water (ddH_2_O). Successively, 0.2 vol% of the dispersing agent Darvan 821-A (Vanderbilt Minerals) was added to the solution and the pH was set to 10.5 through the addition of 1 M sodium hydroxide solution. To reach a Ca/P molar ratio of 1.67, a second solution obtained by dissolving 4.44 g of calcium chloride (Sigma Aldrich, Italy) in 60 mL of ddH_2_O was prepared and, after 1 h of stirring, added dropwise into the solution containing the phosphate precursor while constantly maintaining the pH at 10.5 through the addition of sodium hydroxide. The resulting solution was kept under stirring for 3 h, constantly maintaining the pH at 10.5. After an overnight ageing step performed at atmospheric pressure and room temperature, the supernatant was removed, and the remaining slurry was poured in a 250 mL Teflon-lined hydrothermal reactor and placed in an oven at 100 °C for 4 h. After cooling, the supernatant was removed, and the resulting slurry was centrifuged in order to separate HA particles. The latter were washed three times with ddH_2_O and once with pure ethanol. In the end, nanoHA particles were collected in a Petri dish and dried in an oven at 100 °C for 24 h.

**Protocol for the synthesis of nanoMBG particles adapted from Fiorilli et al** ^35^**.**

Mesoporous bioactive glasses (Ca/Si = 15/85) were synthetized exploiting a base-catalyzed sol–gel method. In details, the calculated amount of cetyltrimethylammonium bromide (CTAB 98%, Sigma Aldrich, Italy) were dissolved in 140 mL of ethanol, 50 mL of ddH_2_O and 25 mL of NH_4_OH (Ammonium hydroxide solution, Sigma Aldrich, Italy) for 30 min under stirring. A second solution containing tetraethyl orthosilicate (TEOS, Tetraethyl orthosili-cate, reagent grade 98%, Sigma Aldrich, Italy) and 50 mL of ethanol was prepared and stirred for 30 min. The TEOS solution was added dropwise into CTAB solution and the resulting mixture was stirred for 20 min. Calcium nitrate tetrahydrate (Ca(NO_3_)2·4H_2_O, 99%, Sigma Aldrich, Italy) was subsequently added and stirred for 10 min. The powders were collected by centrifugation (Hermle Labortechnik Z326) at 10,000 rpm for 3 minutes and washed three times with ddH_2_O, once with 50% ethanol and once with absolute ethanol. The final precipitate was dried at 70 °C overnight and calcined at 600 °C for 5 hours with a heating rate of 1 °C min^-1^ in a Carbolite 1300 CWF 15/5, in order to remove CTAB.

**Supplementary material for Section 2.3**


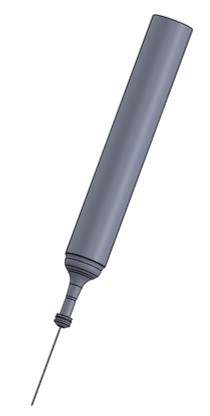


**Figure S1.** CAD model of the fluid inside the printing cartridge and needle, used for the computational simulation.

**Supplementary material for Section 3.1**


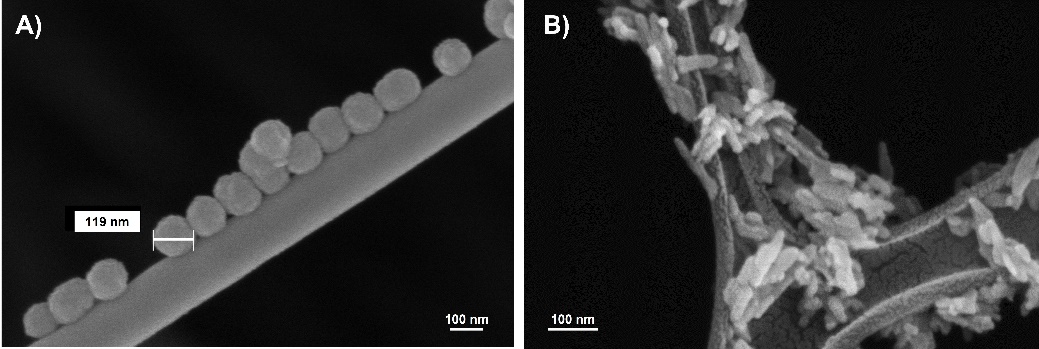


**Figure S2.** FESEM images of nanoMBG (A) and nanoHA (B) particles used to create the hybrid formulation ^31,35^.

**Supplementary material for Section 3.4**


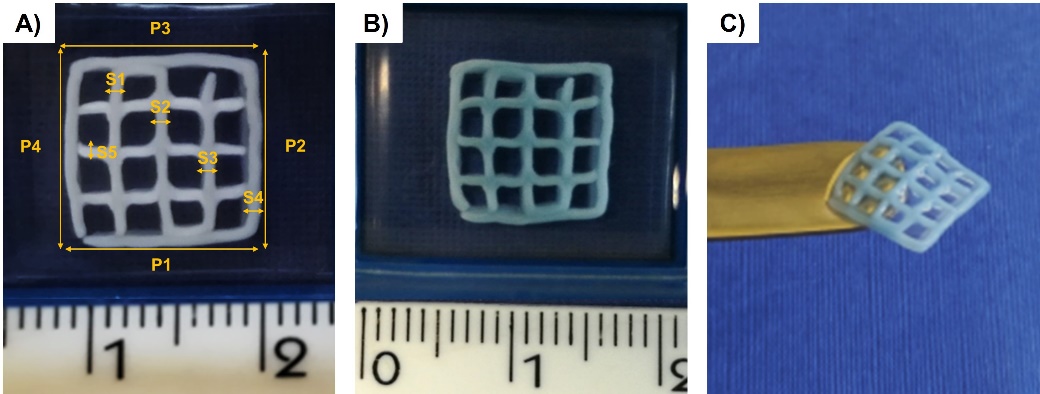


**Figure S3.** Images of reference points of measure considered for all scaffolds (A) and scaffolds printed with the optimized parameters (30 kPa; 3 mm/s) after chemical crosslinking with genipin (B, C).

**Table S1.** Set of measurements obtained from ImageJ analyses for the produced scaffolds.

| Samples | Point of measure | 30 kPa;  2 mm/s  (Fig. 4B) | 30 kPa;  3 mm/s  (Fig. 4F) | 40 kPa;  4 mm/s  (Fig. 4C) | 40 kPa;  5 mm/s  (Fig. 4G) | 50 kPa;  6 mm/s (Fig. 4D) | 50 kPa;  7 mm/s  (Fig. 4H) |
| --- | --- | --- | --- | --- | --- | --- | --- |
| Sides  (mm) | P1 | 11.62 | 11.39 | 16.23 | 11.77 | 13.40 | 9.75 |
|  | P2 | 11.93 | 11.64 | 15.92 | 11.55 | 13.86 | 10.35 |
|  | P3 | 12.37 | 11.82 | 16.30 | 12.27 | 14.26 | 10.50 |
|  | P4 | 12.18 | 11.14 | 15.92 | 11.94 | 14.33 | 10.55 |
| Strand width (mm) | S1 | 0.68 | 0.57 | 0.84 | 0.44 | 0.80 | 0.70 |
|  | S2 | 0.75 | 0.50 | 0.92 | 0.44 | 0.73 | 0.50 |
|  | S3 | 0.68 | 0.46 | 0.92 | 0.38 | 0.80 | 0.50 |
|  | S4 | 0.81 | 0.60 | 1.00 | 0.55 | 0.73 | 0.45 |
|  | S5 | 0.68 | 0.57 | 0.76 | 0.38 | 0.73 | 0.55 |

**Table S2.** Set of measurements obtained from ImageJ analysis for the original CAD file and scaffold printed with the optimized parameters (30 kPa; 3 mm/s) before and after chemical crosslinking.

| Samples | Point of measure | CAD model  (Figure 4E) | Non-crosslinked scaffold  30 kPa; 3 mm/s  (Figure 4F) | Crosslinked scaffold  30 kPa; 3 mm/s  (Figure 5D) |
| --- | --- | --- | --- | --- |
| Sides (mm) | P1 | 9.44 | 11.39 | 10.58 |
|  | P2 | 10.16 | 11.64 | 10.82 |
|  | P3 | 9.47 | 11.82 | 11.23 |
|  | P4 | 10.03 | 11.14 | 11.17 |
| Strand width (mm) | S1 | 0.51 | 0.57 | 0.58 |
|  | S2 | 0.61 | 0.50 | 0.58 |
|  | S3 | 0.45 | 0.46 | 0.58 |
|  | S4 | 0.61 | 0.60 | 0.64 |
|  | S5 | 0.64 | 0.57 | 0.47 |

**Table S3.** Mean values of storage modulus (G’), loss modulus (G’’) and denaturation temperature of Coll/nanoMBG/nanoHA before and after chemical crosslinking with genipin.

| Samples | Storage modulus G’ (Pa) | Loss modulus G’’  (Pa) | Denaturation  Temperature (°C) |
| --- | --- | --- | --- |
| Coll/nanoMBG/nanoHA after physical crosslinking at 37 °C | 369.5 ± 31.4 | 91.6 ± 12.7 | 42.0 ± 0.9 |
| Coll/nanoMBG/nanoHA after chemical crosslinking with genipin | 3100.0 ± 71.8 | 216.2 ± 45.8 | 65.0 ± 1.2 |
